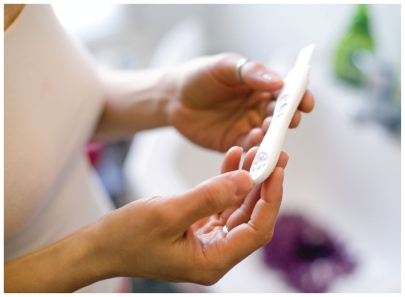# Pregnant Pause: Does Maternal PBDE Exposure Extend Time to Pregnancy?

**DOI:** 10.1289/ehp.118-a217a

**Published:** 2010-05

**Authors:** Julia R. Barrett

**Affiliations:** **Julia R. Barrett**, MS, ELS, a Madison, WI–based science writer and editor, has written for *EHP* since 1996. She is a member of the National Association of Science Writers and the Board of Editors in the Life Sciences

Animal studies indicate polybrominated diphenyl ethers (PBDEs) are endocrine disruptors, potentially affecting the role of thyroid hormones in regulating the reproductive cycle and fertility. The compounds also have been associated with delayed puberty and altered estradiol levels in female animals. Very little is known about the potential effects of PBDEs on human reproductive health, though, and a new study is the first to characterize a specific concern—a relationship between PBDE blood concentrations and a delay in achieving pregnancy **[*EHP*** **118:699–704; Harley et al.]**.

PBDEs are used as flame retardants in furniture, carpeting, textiles, electronics, and plastics. Commercial mixtures of PBDEs contain a variety of congeners, or chemical variations. Data collected by the Centers for Disease Control and Prevention suggest 97% of Americans may have detectable levels of PBDEs in their blood.

The current study included 223 pregnant women enrolled in the Center for the Health Assessment of Mothers and Children of Salinas (CHAMACOS), a longitudinal birth cohort study focused on environmental exposures and reproductive health in California’s Salinas Valley. Upon enrollment, the women reported their reproductive history, previous use of contraception and fertility medication, whether the pregnancy was planned, and how long it took to become pregnant after stopping contraception.

Blood samples collected around 26 weeks of pregnancy were analyzed for 10 PBDE congeners. Statistical analyses focused on those most commonly found: BDE-47, BDE-99, BDE-100, and BDE-153. BDE-100 and BDE-153 were the most strongly associated with longer time to pregnancy. For each month, the likelihood of becoming pregnant was 40% or 50% lower with a 10-fold increase in concentration of BDE-100 or -153, respectively. With a 10-fold increase in the total of all 4 congeners, there was a 30% decrease in the odds of pregnancy each month.

The study relied on self-reported time to pregnancy, which is subject to a number of biases. In addition, the study’s findings are limited to 4 PBDE congeners and may not extend to a broader population. Consequently, further research incorporating more congeners and a more representative population is needed. However, given the likelihood of PBDE exposure in the general population, even a small effect of these chemicals on fertility may affect a large number of individuals.

## Figures and Tables

**Figure:**